# Intracranial pressure and optic disc changes in a rat model of obstructive hydrocephalus

**DOI:** 10.1186/s12868-022-00716-w

**Published:** 2022-05-23

**Authors:** Snorre Malm Hagen, Sajedeh Eftekhari, Steffen Hamann, Marianne Juhler, Rigmor H. Jensen

**Affiliations:** 1 Department of Ophthalmology, Rigshospitalet, University of Copenhagen, Valdemar Hansens Vej 13, 2600 Glostrup, Denmark; 2 Danish Headache Center, Department of Neurology, Glostrup Research Institute, Rigshospitalet, University of Copenhagen, Nordstjernevej 42, 2600 Glostrup, Denmark; 3 Department of Neurosurgery, Rigshospitalet, University of Copenhagen, Blegdamsvej 9, 2100 Copenhagen Ø, Denmark

**Keywords:** Hydrocephalus, Intracranial pressure, Papilledema, Epidural, Kaolin, Fluid-filled system.

## Abstract

**Background:**

The kaolin induced obstructive hydrocephalus (OHC) model is well known for its ability to increase intracranial pressure (ICP) in experimental animals. Papilledema (PE) which is a predominant hallmark of elevated ICP in the clinic has not yet been studied in this model using high-resolution digital fundus microscopy. Further, the long-term effect on ICP and optic nerve head changes have not been fully demonstrated. In this study we aimed to monitor epidural ICP after induction of OHC and to examine changes in the optic disc. In addition, we validated epidural ICP to intraventricular ICP in this disease model.

**Method:**

Thirteen male Sprague-Dawley rats received an injection into the cisterna magna containing either kaolin-Ringer’s lactate suspension (n = 8) or an equal amount of Ringer’s lactate solution (n = 5). Epidural ICP was recorded post-operatively, and then continuously overnight and followed up after 1 week. The final epidural ICP value after 1 week was confirmed with simultaneous ventricular ICP measurement. Optic disc photos (ODP) were obtained preoperatively at baseline and after one week and were assessed for papilledema.

**Results:**

All animals injected with kaolin developed OHC and had significant higher epidural ICP (15.49 ± 2.47 mmHg) compared to control animals (5.81 ± 1.33 mmHg) on day 1 (*p* < 0.0001). After 1 week, the epidural ICP values were subsided to normal range in hydrocephalus animals and there was no significant difference in epidural ICP between the groups. Epidural ICP after 1 week correlated with the ventricular ICP with a Pearson’s r = 0.89 (*p* < 0.0001). ODPs from both groups showed no signs of acute papilledema, but 5 out of 8 (62.5%) of the hydrocephalus animals were identified with peripapillary changes.

**Conclusions:**

We demonstrated that the raised ICP at day 1 in the hydrocephalus animals was completely normalized within 1 week and that epidural ICP measurements are valid method in this model. No acute papilledema was identified in the hydrocephalus animals, but the peripapillary changes indicate a potential gliosis formation or an early state of a growing papilledema in the context of lateral ventricle dilation and increased ICP.

## Introduction

Papilledema refers to optic disc swelling caused by increased intracranial pressure (ICP) and is a vision threatening condition. Increased ICP and papilledema is related to several neurological disorders, e.g., brain hemorrhages, brain ischemia, traumatic brain injury, brain tumors, hydrocephalus and idiopathic intracranial hypertension (IIH) [1]. The complexity of these conditions complicates optimal treatment and prognosis. Increased ICP itself is a condition correlated with high risk of morbidity and mortality [2].

The development of papilledema is caused by increased pressure in the subarachnoid space (SAS) of the brain, which is continuous with the optic nerve sheath. On leaving the eye, the axons of the retinal ganglion cells that constitute the optic nerve enter the optic nerve head (ONH) and traverse the underlying fine-meshed structure known as lamina cribrosa. An elevation in SAS pressure impedes the axoplasmic transport and entails prelaminar axonal swelling in the ONH and peripapillary retina [3]. Currently, the association of disease models of increased ICP and papilledema has not been fully developed in rodents. The kaolin animal models has been widely used to understand cerebrospinal fluid (CSF) dynamics due to the abrupt induction and long-term capabilities either as obstructive or communicating hydrocephalus [4–7].

To our knowledge the retinal and ONH changes in kaolin obstructive hydrocephalus (OHC) animal-models have only been investigated in very few studies [8, 9]. Digital high-resolution fundus microscopes for rodents now make it feasible to visualize and track in vivo changes of the retina and ONH over time. Our group has developed and validated a novel method for long-term epidural ICP recording in rats, and we have shown that this is a less invasive and more reliable alternative to long-term ventricular ICP recording in rats [10, 11].

In this study, we aimed to assess ICP fluctuations for up to 7 days after kaolin induced OHC in rats using an epidural ICP recording system based on the fluid-filled setup. In addition, we validated the epidural recording to intraventricular recording in the same animals. Furthermore, we assessed for development of optic disc edema and/or retinal changes related to the increased ICP.

## Materials and methods

### Animals

Thirteen male Sprague-Dawley (SD) rats (Taconic Bioscience Inc., Denmark) with a mean weight of 350 g were used. The rats were housed in groups of 4 with access to ad libitum standard rodent diet and water in animal facilities (Glostrup Research Institute, Rigshospitalet) with controlled temperature, humidity and an inverted 12-hour light/dark cycle. The study was approved by the Institutional Animal Care and Use Committee (Glostrup Research Institute) and accomplished in compliance with Danish law. License (2012-15-2934-00283) granted by the Danish Animal Experiments Inspectorate. All methods and data are reported according to ARRIVE guidelines.

### Medication

Invasive procedures and eye exams were performed under anesthesia induced with 2.7mL/kg (maintained with 1.0mL/kg every 30 min) of balanced anesthetic mixture (1.25 mg/mL Midazolam (Hameln Pharmaceuticals) and “Hypnorm” with 2.5 mg/mL fluanisone and 0.079 mg/mL fentanylcitrate (Skanderborg Farmacy) injected subcutaneously (s.c.).

On the day of surgery and the following 2 days, anti-inflammatory (5 mg/kg carprofen, Rimadyl®, Pfizer), antibiotic (10 mg/kg enrofloxacin, Baytril®, Bayer) and analgesic (0.03 mg/kg buprenorphine, Temgesic®, RB Pharmaceuticals) treatment was injected s.c. For euthanasia an intraperitoneal injection of a pentobarbital-lidocaine mixture (65 µL/kg) was used.

### Study design

Rats were randomly assigned into 2 groups. Eight animals in the hydrocephalus group were injected with a suspension of kaolin into the cisterna magna and 5 animals in the control group was similarly injected with an equal amount of the vehicle (Ringer’s lactate solution). Optic disc photo (ODP) was obtained preoperatively at day 0 and again after 7 days to allow detection of in vivo changes.

Epidural ICP was recorded at day 0 postoperatively and continuously until day 1 for approximately 18–22 h and then again at day 7. In all animals the final epidural ICP recording was also followed by an intraventricular ICP recording to validate the epidural ICP. The intraventricular recording was performed via the prepositioned guide probe installed epicranially at day 0 and therefore the dura and ventricle were not penetrated until day 7. Puncturing the dura and canulating the ventricles were deliberately avoided initially at day 0 to avoid neuro-infection and CSF shunting. In the end of the study, animals were sacrificed with a pentobarbital-lidocaine mixture and the brains were removed immediately and fixed overnight in paraformaldehyde (4%) in phosphate buffered saline (PBS) at 4 °C.

### Induction of hydrocephalus

The anesthetized rat was placed on a heating pad and the head was fixed in a stereotactic frame (David Kopf Instruments). With the neck flexed in 90 degrees angle the rhomboid atlanto-occipital membrane above the cisterna magna was marked on the skin between the skull and the first cervical spinosus. An insulin syringe with a 30 G needle was filled with either 90 µL of a 25% sterile kaolin suspension (in vehicle) or 90 µL of the vehicle alone. Ringer’s lactate solution (1.4 mM Ca^2+^, 4 mM K^+^, 130 mM Na^+^, 109 mM Cl^−^, 28 mM lactate) was used as vehicle. The needle was placed in the previously marked area and slowly advanced in a vertical direction until there was a loss of resistance. The percutaneous injection was performed gradually over a period of 10 s. Following the injection, the neck was extended, the upper jaw fixed in the stereotactic frame and the rat was observed for respiratory depression.

### Implantation of epidural probe and ventricular guide probe

Surgical procedures and ICP recordings was carried out similar to the method described in detail by Uldall et al. [10]. The method is described in brief below.

Local anesthesia and vasoconstrictor (10 mg/mL lidocaine and 5 µg/mL) were injected subcutaneous in the midline of the scalp. A 2 cm midline incision was made with a scalpel from just above the eyes to between the ears. The underlying fascia was separated with forceps and a wound retractor was inserted. The remaining fascia and soft tissue were removed until bregma, lambda and the frontal and partial bones were exposed. Carefully, to avoid any damage or penetration of the dura mater, 5 holes were made with a dental drill; 1 for the epidural probe (C313G-3UP, PlasticsOne®), 1 for the ventricular guide probe (C313G-0-0.4, PlasticsOne®) and 3 for anchoring screws. The probes were anchored to the anchoring screws with light-curing dental resin cement (Panavia™ SA Cement Plus). The epidural probe was protected by a cap (303DCFT-1-GFN, PlasticsOne®) sealed with dental rubber cement. The ventricular guide probe was protected by a cap (C313DC-GFN-SP, PlasticsOne®) with an internal metal dummy cannula cut 1 mm below the pedestal of the probe, also sealed with dental rubber cement. The incision of the skin was closed with inverted sutures (4−0 Vicryl™ Plus, Ethicon) and covered with vaseline.

### ICP recordings

Prior to epidural ICP recordings the epidural probe was cleaned and flushed with an internal cannula (C311IU cut in level with the pedestal of C313G-3UP, PlasticsOne®) before the tube was connected to the epidural probe.

For the single intraventricular ICP recording the internal cannula (C311I cut 5 mm below the pedestal of C313G-0-0.4, PlasticsOne®) was slowly advanced in the ventricular guide probe until a pulsating drop of CSF was observed on top. The tube was then connected to the internal cannula and held in place with an outer tubing having a captive collar (C313C, PlasticsOne®).

Proper ICP trace was confirmed by performing a bilateral jugular vein compression, which transiently raised the ICP. ICP data from day 0, 1 and 7 was collected as a 5-minute mean preceded by 10 min of stable ICP reading. Continuous ICP data was collected from each animal as a 5-minute mean for every 30 min of recording.

### Optic disc photography

ODP was obtained from both the hydrocephalus group and the control group preoperatively at day 0 and after 7 days. ODPs were captured with a digital fundus microscopy system (Phoenix Micron IV In Vivo Imaging Microscope, StreamPix v5.19, Phoenix Research Labs) designed for retinal imaging of laboratory rodents.

Rats were anesthetized and immediately after administered pupil dilating drops (1 drop/eye tropicamide 0.5%) and placed on an adjustable stage. Pupil dilation was determined by directing the light from the image microscope towards the eyes. Coupling gel (1–2 drops/eye GoniSoft ®) was applied to protect and moist the cornea while the lens was advanced to the eye. The optic disc and peripapillary retina were observed, and an images sequence was recorded for 10–15 s with different light intensity and focus point for both eyes.

Fundus recordings were evaluated in Micron Discover for Phoenix Micron IV In Vivo Imaging Microscope v1.2 and the highest quality ODPs were saved as bitmap image files.

General signs of papilledema were defined as one or more of following: changes to the optic disc margin, peripapillary halo, vessel engorgement and/or torsion and/or obscurations, hemorrhages, peripapillary choroidal folds. These papilledema signs were used as basis for a blinded evaluation by an experienced neuroophthalmologist (SH). Day 0 ODPs and day 7 ODPs from each individual animal were compared and either marked as “no optic disc changes” or “optic disc changes”. Animals with unilateral or bilateral changes were both classified as having “optic disc changes”. For the animals assessed with “optic disc changes” the changes included paleness in the temporal upper quadrant of the optic disc margin and the peripapillary area.

### Validating hydrocephalus

Kaolin induced OHC can be detected anatomically with significantly dilated lateral ventricles at day 7 after successful kaolin injection [5, 12]. To confirm the disease model, gross anatomical changes was assessed by cutting the brains in 1 mm coronal slices and midbrain slices was photographed to confirm ventricle changes.

### Statistical analysis

Sample sizes were calculated with a statistical power analysis (alpha = 0.05, power 0.80) and enrolled 2:1 based on a pilot study where 25% were sacrificed because of adverse effects on hydrocephalus induction. All ICP values are presented as mean ± standard deviation (SD) if not stated otherwise. ICP data were collected from Perisoft for Windows v2.5.5 and visualized in GraphPad Prism 9 using two-way ANOVA for group comparison. Linear regression analysis was performed in GraphPad Prism 9 and normal distribution was ensured using Shapiro-Wilk test. The Banard’s exact test was performed for group comparison of optic disc changes in Rstudio v4.1.0 using the Barnard-package v1.8. *p*-values below 0.05 was considered significant.

## Results

### ICP

Baseline epidural ICP recordings were obtained for all animals post operatively at day 0. One animal from the control group lost ICP trace few hours into the continues recording and was excluded from the ICP data. In the hydrocephalus group, the mean baseline epidural ICP value was 3.17 ± 1.23 mmHg. The mean baseline epidural ICP value in the control group was 2.26 ± 0.34 mmHg and there was no difference between the groups (*p* > 0.99) (Table 1).

**Fig. 1:**
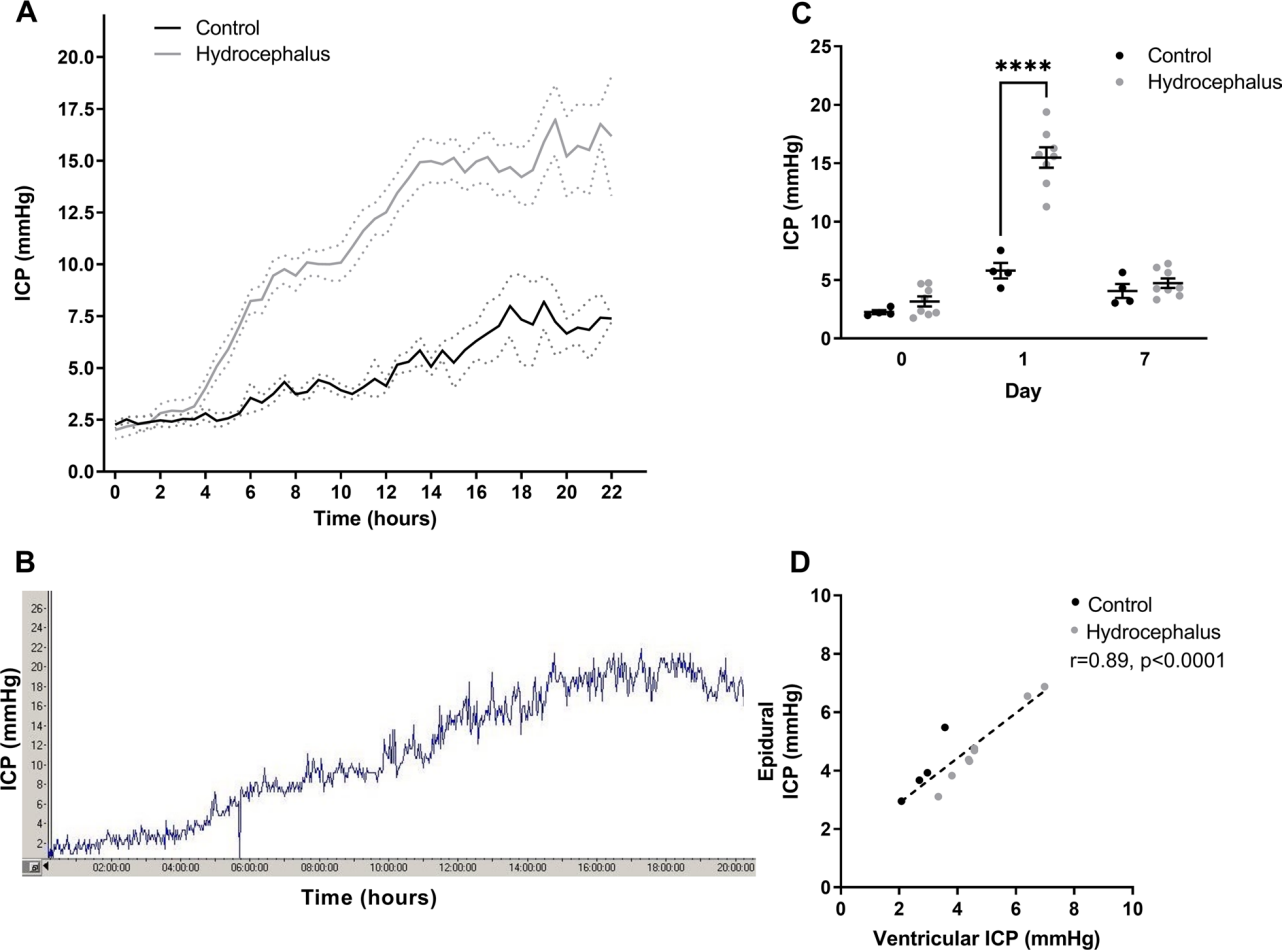
Acute epidural intracranial pressure (ICP) elevation in obstructive hydrocephalic (OHC) animals compared to control animals. **A** Mean curves showing the postoperative mean continuous epidural ICP (dots representing ± SEM). **B** Raw ICP trace from the overnight continuous recording in an OHC animal (X-axis in hours and Y-axis in mmHg). **C** Epidural ICP values from each animal in both groups are plotted at day 0, 1 and 7 (mean is showed with SEM error bars, **** = p < 0.0001). **D** Epidural ICP correlated with the ventricular ICP values at day 7 (Pearson’s r = 0.89, *p* < 0.0001)

The continuous recordings showed a significant rise in epidural ICP in all hydrocephalus animals within the first 6 h and continued over night until day 1 and only a minor rise in epidural ICP was seen in the control group (Fig. 1A). A raw ICP trace after injection of kaolin is displayed in Fig. 1B.

Day 1 ICP values were measured approximately 18 to 22 h post-operatively and the epidural ICP of the hydrocephalus animals remained significant higher compared to control animals (*p* < 0.0001) (Fig. 1C). On day 7, the epidural ICP in the hydrocephalus animals were returned to physiological values and there was no significant difference between the two groups (*p* > 0.99) (Fig. 1C).

The epidural ICP values before and after ventricular cannulation correlated with the ventricular ICP recordings on day 7 (Pearson’s r = 0.89, *p* < 0.0001) (Fig. 1D).

**Table 1:** Mean intracranial pressure (ICP) values measured at baseline, day 1 and day 7

Group	Baseline ICP mean ± SD [min, max]	Day 1 ICP mean ± SD [min, max]	Day 7 ICP, mmHg mean ± SD [min, max]		
	Epidural, mmHg	Epidural, mmHg	Epidural, mmHg	Epidural after ventricular cannulation, mmHg	Ventricular, mmHg
Hydrocephalus *n* = 8	3.17 ± 1.23 [1.76, 4.76]	15.49 ± 2.47 [11.28, 19.38] ****	4.74 ± 1.15 [3.33, 6.42]	4.81 ± 1.25 [3.34, 6.98]	4.82 ± 1.29 [3.11, 6.88]
Control *n* = 4	2.26 ± 0.34 [1.98, 2.75]	5.81 ± 1.33 [4.32, 7.55]	4.06 ± 1.20 [3.05, 5.65]	4.01 ± 1.07 [2.95, 5.48]	2.83 ± 0.31 [2.08, 3.57]

Obstructive hydrocephalus animals showed statistically significant elevations of the epidural ICP day 1 after kaolin injection compared to control animals. At day 7 the epidural ICP was measured before intraventricular cannulation and then simultaneously with the ventricular ICP to compare the two recording sites. Comparison ****p < 0.0001

### Optic disc photos

**Fig. 2:**
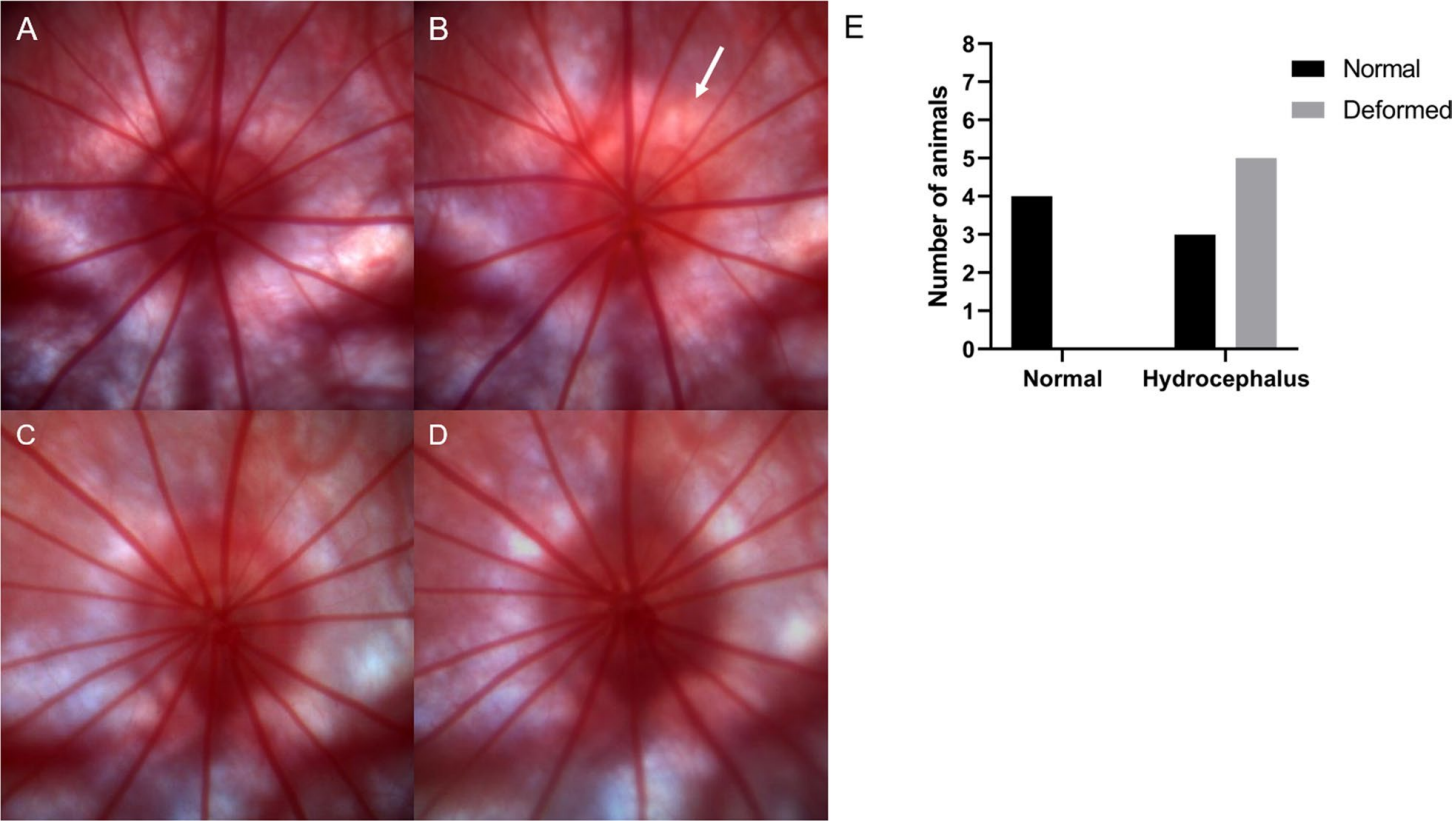
Optic disc photos showing peripapillary changes in an obstructive hydrocephalic (OHC) animal. **A** + **B** ODP from a left eye displaying a normal optic disc at baseline (**A**) and after 1 week with OHC (**B**). White arrow is pointing out paleness in the upper temporal peripapillary area. **C** + **D** ODP from a left eye displaying a normal optic disc at baseline and after 1 week after sham injection. No considerable changes were observed. **A**–**D** Left eyes are illustrated in this figure, but both eyes were evaluated in all animals. **E** Bar diagram presenting distribution of normal and deformed optic discs in context of hydrocephalus

One animal from the control group was excluded because of bilateral corneal damage at day 7, which made it impossible to evaluate the ONH and retina. Two animals had partial corneal damage in one of the eyes but were not excluded as fundus examination with good technical quality was still possible. Hence, 48 ODPs from 12 rats were selected for blinded evaluation. Five of 8 animals (62.5%) in the hydrocephalus group were assessed to have optic disc changes on day 7 compared to baseline ODPs (unilateral (n = 2), bilateral (n = 3)) (Fig. 2A, B, E). In the control group, no changes were observed in the optic disc or the peripapillary area compared to baseline ODPs (Fig. 2C, D, E). A relationship between enlarged lateral ventricles and optic disc changes was observed (*p* = 0.04). However, none of the ODPs showed any ONH protrusion, vessel engorgement, vessel obscurations, hemorrhages, choroidal folds or microhemorrhage indicating acute papilledema.

### Validating hydrocephalus

**Fig. 3:**
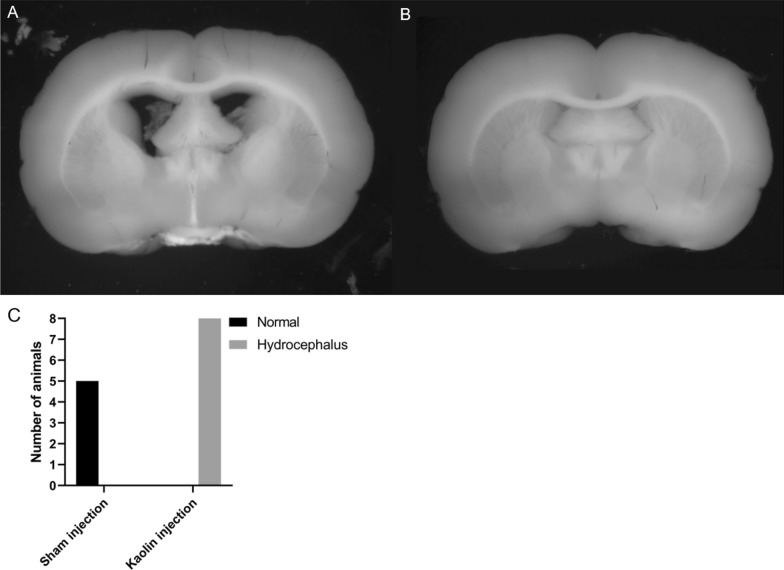
Ventricular dilation after kaolin insertion in the obstructive hydrocephalic (OHC) animal. **A** Midbrain slice showing ventricular dilation in an OHC animal 1 week after kaolin insertion. Note the deposition of kaolin at the base of the brain. **B** Midbrain slice of a control animal 1 week after sham insertion. **C** Bar diagram presenting distribution of normal and enlarged lateral ventricles in sham (control group) and kaolin (hydrocephalus group) injected animals

To confirm proper kaolin deposition a microscopic inspection of the neck and brain was performed. Animals in the hydrocephalus group revealed expected kaolin deposition in the cisterna magna and ventral to the brainstem. There was only a small amount of kaolin in the needle duct at the injections site, which ensured successful kaolin injections. Hydrocephalus was confirmed by showing enlargement of the lateral ventricles in the midbrain slices (Fig. 3A). This was observed in all animals in the hydrocephalus group compared to none of the animals in the control group (Fig. 3B, C).

## Discussion

In the clinical setting, structural damage to the visual afferent system associated with impaired visual function is a disabling consequence of raised ICP. In OHC and other conditions with raised ICP, edema of the optic disc is a predominant finding [13, 14]. Early treatment can restore normal ONH integrity and visual function and can be achieved by treating the underlying cause (e.g., surgical removal of tumors) or be directed on decreasing ICP (e.g., CSF diversion (shunting) or reduction of CSF production (medication).

To our knowledge this is the first study to observe in vivo optic disc changes in context of increased ICP and obstructive hydrocephalus in rats.

### Induction of hydrocephalus and ICP elevation

In this study we have demonstrated that ICP in the kaolin OHC rats significantly increases during the first day after induction compared to control rats. In the same hydrocephalus rats we showed that epidural ICP subsides to normal range after one week, confirmed by intraventricular ICP.

All animals included in this study had baseline epidural ICP values similar to previous studies [10, 11, 15, 16]. Previously we showed that the physiological epidural ICP in normal SD rats is around 5 mmHg corresponding to both hydrocephalus and control group at day 7 in this study [11]. The method used for injecting kaolin into the cisterna magna was safe and effective. The injected volume of 90 µL (25% w/v) used in this study was in the upper range of published amounts for production of obstructive hydrocephalus (30–100 µL, 20–25% w/v). We used this higher volume to effectively increase ICP and development of hydrocephalus [17]. All animals in the hydrocephalus group successfully developed OHC demonstrated by having enlarged lateral ventricles after 1 week.

Previous studies have shown that the kaolin induced OHC model in rodents increases ICP [7, 18–22]. However, only a few have studied ICP within the first days after induction of OHC [18, 19], and only two in OHC rats [20, 21]. We found the same ICP fluctuation within the first week of OHC with a significant increase in ICP in the first 22 h. This finding is comparable to a study with 48 h continuous telemetric ICP recordings in 3 kaolin induced OHC rats using the same epidural recording site [20]. The same study also indicates that the ICP gradually subsides after 24–48 h, which substantiates our findings after one week. Experiments in mice and hamsters show the same immediate increase in ICP the first day after kaolin insertion but here ICP increase to higher values after 1 week [18, 19].

In this study the normalization of the ICP observed in the hydrocephalus animals are comparable with the clinical condition “arrested hydrocephalus (AH)/compensated hydrocephalus”. AH is a compensatory physiological state where the CSF production and absorption has met an equilibrium with ICP in normal range, but the underling mechanisms are not fully described. We assume that the decline in ICP observed in our study could be explained by the dilation of the ventricle system and a physiological adaption (e.g. changes in aquaporin-4 distribution [5, 18] and forming of accessory CSF-pathways [23, 24]) as hypothesized in AH [25]. Based on a prior study where ICP in OHC rats rose after 4 weeks from kaolin injection [7], this current study could also indicate that there may be a bimodal increase in ICP in the kaolin induced OHC rat model. Future studies, in which ICP is measured in OHC animals continuously in an extended period will help to clarify this hypothesis.

### Fundoscopic changes

We found that more than half (62.5%) of the kaolin induced OHC rats developed a pale peripapillary change following a short period of increased ICP. This in vivo finding has not been described earlier in a rat model of increased ICP. It is a significant finding highly relevant for future research in understanding of papilledema development when modelling hydrocephalic conditions with increased ICP. We found no signs of any optic disc obscuration, vessel engorgement/torsion/obscurations, hemorrhages or peripapillary choroidal folds representing papilledema as seen in humans with acute papilledema. Fundoscopic visible papilledema in rats has, to the best of our knowledge, only been visualized in vivo in a rat model of anterior ischemic optical neuropathy [26]. Early studies state that a growing cerebral tumor in rats can result in the development of papilledema and vessel engorgement, but not by inducing OHC injecting kaolin in the cisterna magna alone [9]. Conversely, it has been demonstrated that the optic nerve of neonatal rats with kaolin induced OHC undergoes morphometric changes with a delay on myelination, and the rhesus monkey with kaolin induced OHC successfully developed papilledema [27, 28]. The relatively short, < 1 week, duration of ICP elevation in our study may explain why we did not see the characteristics of an acute papilledema at day 7. Further, we have not investigated the animals in the first days where the ICP was markedly increased so transient papilledema cannot be excluded here. Another potential explanation could be the less developed lamina cribosa structure discovered in rats, which might limit axonal stasis and papilledema development[29]. Although rats have a fully developed visual system, it is far less complex (100,000-110,000 axons in the rat optic nerve [30]) than in primates (> 1 million axons in the rhesus monkey optic nerve [31]), which can account for the altered visual appearance of papilledema in rodents. We suggest two hypotheses for the pale peripapillary change observed in the OHC animals: (1) An expression of a prodromal stage in the development of papilledema representing a halo that partially covers the circumference of the optic disc. (2) A remission of a mild papilledema with subtle gliosis formation.

Considering the small numbers of animals in this study our findings remains an indication and more data are needed to confirm this.

### Limitations

Injecting substances into the brain may be associated with risk of some complications. As injection of substances into the cisterna magna can lead to, acute respiratory depression and sudden death, the kaolin injection in the current study was performed before the implantation of the ICP probes. The baseline ICP was therefore performed after the kaolin injection, which means that any increase in ICP due to the procedure of the injected volume, could not be measured.

Ideally the intraventricular ICP should be measured simultaneously with the epidural ICP at all time points. To avoid the risk of hydrocephalus and increased ICP due to infection associated with intraventricular cannulation, the intraventricular ICP recordings were only performed at day 7. Previously, we have demonstrated a very clear relation between the epidural and intraventricular ICP in our prior validation study, and not unexpected, we found a very high complication rate with intraventricular ICP recording[10].

To evaluate the optic disc, it is crucial to maintain a transparent cornea. In this study, we had to exclude 1 control animals with bilateral corneal damage, which means that the natural variability of the optic disc over time is difficult to outline. In long-term experiments where the animal is anesthetized several times, there is a risk of corneal damage and clouding. Therefore, it is important to keep the cornea moist with artificial teardrops during recovery. Capturing an ODP at different time points is also associated with some variance due to several technical factors (e.g., light intensity and fundus microscope lens-to-papilla distance). As the OHC animals are fragile and not capable to receive further anesthesia during the first week after kaolin administration and surgery, no ODPs were taken between day 1–6. Thereby, potential papilledema at this stage might therefore have been missed. For quantification of the optic disc changes, future studies with ODP and optical coherence tomography (OCT) within the first few days after induction of increased ICP are required. This can be achieved by terminate the study earlier or perform the study without ICP surgery. Quantification of the optic disc volume and retinal fiber layer thickness using OCT and histology could support the peripapillary findings on the ODPs and show subtle changes not visible on the ODPs.

## Conclusions

This study demonstrated that the ICP in the kaolin induced OHC rat model gradually increased the first day after induction but normalized after 1 week simulating the clinical condition AH. Further, we identified that the epidural ICP recording method in a rat model of increased ICP was very valid and less invasive. Another interesting finding was the peripapillary changes seen after 1 week in the rats exposed to increased ICP indicating a prodromal change to papilledema or potentially a mild papilledema in remission with subtle gliosis formation. Further investigations of the ONH and neuroretina using OCT and histology along with long-term continuous ICP recording in awake animals are needed.

## Data Availability

Data generated or analyzed during the current study are available from the corresponding author on reasonable request.

## Abbreviations

ICP: Increased intracranial pressure.
SAS: Subarachnoid space.
ONH: Optic nerve head.
CSF: Cerebrospinal fluid.
OHC: Obstructive hydrocephalus.
SD: Sprague-Dawley.
ODP: Optic disc photo.
PBS: Phosphate buffered saline.
AH: Arrested hydrocephalus.
OCT: Optical coherence tomography.
